# Colchicine in cardiac disease: a systematic review and meta-analysis of randomized controlled trials

**DOI:** 10.1186/s12872-015-0068-3

**Published:** 2015-08-29

**Authors:** Subodh Verma, John W. Eikelboom, Stefan M. Nidorf, Mohammed Al-Omran, Nandini Gupta, Hwee Teoh, Jan O. Friedrich

**Affiliations:** Division of Cardiac Surgery, Keenan Research Centre for Biomedical Science and Li Ka Shing Knowledge Institute of St. Michael’s Hospital, Toronto, ON Canada; Division of Vascular Surgery, Keenan Research Centre for Biomedical Science and Li Ka Shing Knowledge Institute of St. Michael’s Hospital, Toronto, ON Canada; Division of Endocrinology & Metabolism, Keenan Research Centre for Biomedical Science and Li Ka Shing Knowledge Institute of St. Michael’s Hospital, Toronto, ON Canada; Department of Surgery, Keenan Research Centre for Biomedical Science and Li Ka Shing Knowledge Institute of St. Michael’s Hospital, Toronto, ON Canada; Department of Medicine, Keenan Research Centre for Biomedical Science and Li Ka Shing Knowledge Institute of St. Michael’s Hospital, Toronto, ON Canada; Department of Critical Care, Keenan Research Centre for Biomedical Science and Li Ka Shing Knowledge Institute of St. Michael’s Hospital, Toronto, ON Canada; Department of Surgery, University of Toronto, Toronto, ON Canada; Department of Medicine and Interdepartmental Division of Critical Care, University of Toronto, Toronto, ON Canada; Department of Medicine, McMaster University, Hamilton, ON Canada; Heart Research Institute, Perth, WA Australia

**Keywords:** Colchicine, Cardiovascular disease, Meta-analysis

## Abstract

**Background:**

Colchicine has unique anti-inflammatory properties that may be beneficial in various cardiovascular conditions. This systematic review and meta-analysis of randomized controlled trials (RCTs) examines this issue.

**Methods:**

We searched MEDLINE, EMBASE, and the Cochrane Database from inception to June 2014 for RCTs using colchicine in adult patients with cardiac diseases. Results were pooled using random effects.

**Results:**

15 RCTs (n = 3431 patients, median treatment 3 and follow-up 15 months) were included. All but 2 used colchicine 1 mg/day. In 5 trials, n = 1301) at risk for cardiovascular disease (coronary artery disease, acute coronary syndrome or stroke, post-angioplasty [2 RCTs], or congestive heart failure), colchicine reduced composite cardiovascular outcomes by ~60 % (risk ratio [RR] 0.44, 95 % confidence interval [CI] 0.28-0.69, p = 0.0004; I^2^ = 0 %) and showed a trend towards lower all-cause mortality (RR 0.50, 95 % CI 0.23-1.08, p = 0.08; I^2^ = 0 %). In pericarditis or post-cardiotomy, colchicine decreased recurrent pericarditis or post-pericardiotomy syndrome (RR 0.50, 95 % CI 0.41-0.60, p < 0.0001; I^2^ = 0 %; 8 RCTs, n = 1635), and post-pericardiotomy or ablation induced atrial fibrillation (RR 0.65, 95 % CI 0.51-0.82, p = 0.0003; I^2^ = 31 %; 4 RCTs, n = 1118). The most common adverse event was diarrhea. Treatment discontinuation overall and due to adverse events (RR 4.34, 95 % CI 1.70-11.07, p = 0.002; I^2^ = 29 %; 7 RCTs, 83/790 [10.5 %] vs. 11/697 [1.6 %]) was higher in colchicine-assigned patients.

**Conclusions:**

Current RCT data suggests that colchicine may reduce the composite rate of cardiovascular adverse outcomes in a range of patients with established cardiovascular disease. Furthermore, colchicine reduces rates of recurrent pericarditis, post-pericardiotomy syndrome, and peri-procedural atrial fibrillation following cardiac surgery. Further RCTs evaluating the potential of colchicine for secondary prevention of cardiovascular events would be of interest.

## Background

Colchicine is used to treat gout and other inflammatory diseases such as familial Mediterranean fever and Behçet’s syndrome [1–6]. Randomized controlled trials (RCTs) have evaluated colchicine in a wide spectrum of cardiac disease [7]. There is now tantalizing evidence that it may prove to be a useful adjunct to current therapy in the treatment and prevention of pericarditis, post procedural atrial fibrillation, atherosclerosis, and stent related disease. In addition, new insights into the role of cholesterol crystal-induced, neutrophil-mediated inflammation in atherosclerosis add plausibility to the clinical benefits observed with its use in secondary prevention of cardiovascular disease [8, 9]. Consequently, we set out to conduct a meta-analysis of RCTs to evaluate the benefits of colchicine in patients with both coronary artery and other cardiac diseases.

## Methods

### Data sources

**Fig 1.**
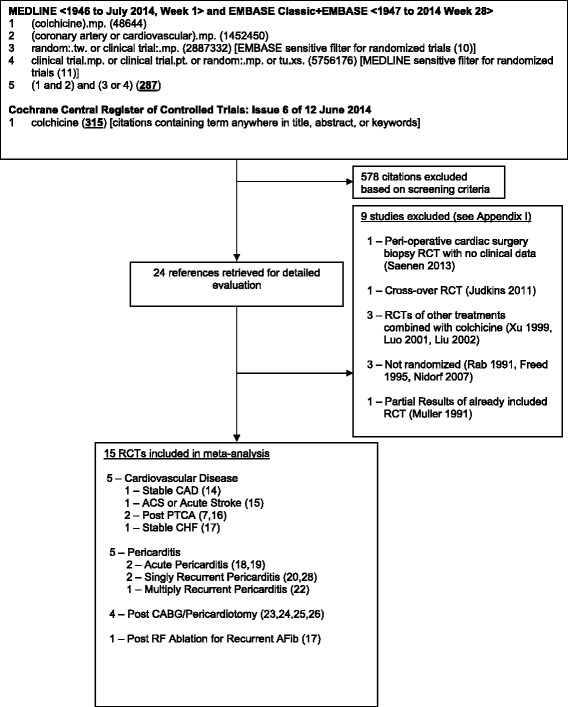
Search Strategy and Trial Flow. Flow chart for the systematic review and meta-analysis showing the search strategy, and the number of studies retained and number of studies excluded with reason for exclusion at each stage of the study selection process. For description of excluded studies see Table 5

We systematically searched OVID versions of MEDLINE (1946 through to July 2014, week 1), EMBASE Classic and EMBASE (1947 through 2014 week 28), and the Cochrane Central Register of Controlled Trials (Issue 5, June 2014) for relevant studies using “colchicine” and “coronary artery” or “cardiovascular” search terms, and previously-published sensitive filters to identify randomized controlled trials (RCTs) [10, 11] (see Fig. 1 for detailed search strategy). We also searched bibliographies of included studies and personal files. We did not impose language restrictions.

### Study selection

We included prospective clinical trials randomizing patients to colchicine vs. placebo or no therapy. Eligible studies included adult patients with cardiac disease including cardiovascular disease, cardiomyopathy or congestive heart failure, pericardial disease, or arrhythmias. Studies were excluded if patients were not randomized between treatment groups. Citations were screened and full text review was conducted to determine eligibility when a screening reviewer felt a citation potentially met inclusion criteria.

### Data extraction and quality assessment

Details of the publication (i.e. trial authors and acronym, enrolment period, year of publication), inclusion/exclusion criteria, demographics and cardiac risk factors of the enrolled patients, description of the interventions used, and outcome definitions and events were collected and collated. Risk of bias in RCTs (including blinding of participants, method of sequence generation and allocation concealment, intention-to-treat analysis, early trial stopping for efficacy before the planned enrollment was completed, and loss to follow-up) was also assessed.

### Data analysis

The primary outcome was the composite cardiovascular outcome as defined by each RCT enrolling patients with cardiovascular diseases, and was recurrent pericarditis or post-pericardiotomy syndrome for RCTs enrolling patients with pericarditis or post pericardiotomy. (Given the different cardiac diseases in the included trials, we only pooled data for trials enrolling patients with relevant conditions for each outcome). Secondary outcomes were all-cause mortality, acute coronary syndrome or non-fatal myocardial infarction, revascularization, and non-fatal stroke (cardiovascular disease trials); and arrhythmias (pericarditis, post-pericardiotomy syndrome, and post radiofrequency ablation trials). Adverse events, and treatment discontinuation data were pooled from all trials as we felt these may be independent of patient condition and to provide the most robust estimate of overall adverse event rates. All analyses were performed using Review Manager (RevMan version 5.2; Cochrane Collaboration, Oxford, UK). Random effects models [12] which incorporate between-trial heterogeneity and give wider and more conservative confidence intervals (CI) when heterogeneity is present were used for all analyses. Statistical heterogeneity among trials was assessed using the I^2^ statistic, defined as the percentage of total variability across studies attributable to heterogeneity rather than chance, and using published guidelines for low (I^2^ = 25 %-49 %), moderate (I^2^ = 50 %–74 %) and high (I^2^ ≥ 75 %) heterogeneity [13]. Relative risks (RR) were used to pool outcomes with a two-sided significance level of 5 %. Individual trial and summary results are reported with 95 % CIs. For the pericarditis RCTs, we use Z-tests of interaction to calculate interaction p-values comparing RRs between separate subgroups (e.g. acute pooled RR vs. recurrent pericarditis pooled RR, etc.). To assess for publication bias, a funnel plot comparing effect measure to study precision was examined for evidence of asymmetry.

## Results

The initial search strategy yielded 287 citations from MEDLINE and EMBASE, and 315 citations from Cochrane, of which 24 were retrieved for full text review. Fifteen RCTs enrolling 3431 patients met inclusion criteria (Fig. 1). Five RCTs measured cardiovascular outcomes in patients with cardiovascular disease including stable coronary artery disease (CAD) [14]. acute coronary syndrome or stroke [15]. post-angioplasty with balloon [16] or bare metal stents [7]. and stable congestive heart failure [17]. Five RCTs enrolled patients with either acute pericarditis [18, 19], recurrent pericarditis [20, 21], or multiply recurrent pericarditis [22]. Four RCTs enrolled patients post-cardiac surgery pericardiotomy [23–26], and 1 RCT post-radiofrequency ablation for atrial fibrillation [27]. Results from two of these RCTs [24, 27] were each published in two publications [28, 29].

### Description of included studies and quality assessment

All but 2 RCTs used a colchicine dose of 1 mg/day. Almost half the trials, mainly those enrolling patients with pericarditis [18–22] or post cardiac surgery [24, 25], reduced the dose to 0.5 mg/day in patients with signs of medication intolerance or body weight <70 kg. Median treatment duration was 3 months. Patient follow-up ranged from 7 days to 3 years (median 15 months).

Tables 1 and 2 provide details of the RCTs including baseline patient characteristics. For the 5 pericarditis RCTs [18–22] mean age was lower (range 48–57 years old) and a lower proportion were male (range 35-60 %) compared to the non-pericarditis RCTs (mean age 57–68 years old, and proportion male 65-89 %). For the non-pericarditis RCTs, patients had expected prevalence of various coronary risk factors including hypertension, diabetes, dyslipidemia and smoking. Previous myocardial infarction or coronary artery disease ranged from 7-40 % in the 7 RCTs that provided this information [14, 15, 21–24, 27]. Patients with congestive heart failure were generally not enrolled except for the one RCT that specifically enrolled such patients [17]. All trials excluded patients with severe renal failure.

**Table 1:** Trial and Baseline Patient Characteristics, and Interventions of RCTs Measuring Cardiovascular Outcomes

	Stable CAD	ACS (91 %) or Acute Stroke (9 %)	Post Successful Elective Balloon PTCA	Post BMS PTCA in DM (31 % ACS)	Symptomatic stable CHF with LVEF ≤ 40 %
Colchicine Dose	0.5 mg/d	1 mg/d	0.5 mg bid	0.5 mg bid	0.5 mg bid
Trial	Nidorf 2013 [14]	Raju 2012 [15]	O’Keefe 1992 [16]	Deftereos 2013 [7]	Deftereos 2014 [17]
	N = 532	N = 80	N = 197	N = 222	N = 267
*Trial Characteristics*					
No. Centres	1	1	1	1	1
Enrolment period	Aug 2008 – May 2010	Apr 2008 – Aug 2009	n/r	n/r	n/r
Treatment/Follow Up	2 (all)/3 (median) yrs	32 days (median)	5.5 months (mean)	6 months (?all)	6 months (all)
Funding	None	Public	n/r	n/r	n/r
*Patients*	N = 532	N = 80	N = 197	N = 196	N = 279
Mean Age (years)	66	57	60	64	67
% Male	89 %	88 %	86 %	65 %	67 %
BMI				27	26
Diabetes	31 %	16 %	12 %	100 %	17 %
HTN		42 %		49 %	36 %
Smoker	5 %	44 %		38 %	
Dyslipidemia		48 %	Total Chol 211 mg/dL		33 %
Prev MI/UA	23 %	18 %			
Prev stroke/TIA		4 %			
PVD		5 %			
CRD	n/r	Excl CrCl <50 mL/min	Excl Cr ≥2.5 mg/dL/ 221 μM	33 % (Excl CrCl <20 mL/min)	Excl eGFR <30 mL/min
Mean LVEF				56 %	28 %
Previous CABG	19 % (Prev PCI 58 %)		26 %		
*Medications*					
ASA and/or clopidogrel	93 % (DAPT 12 %)	100 % (DAPT 85 %)			
Statin	95 %	98 %			63 %
Beta-Blocker	67 %				79 %
Calcium Channel Blocker	14 %				
ACE Inhibitor	58 %				85 % (incl ARB)
Diuretic					69 %

**Table 2:** Trial and Baseline Patient Characteristics, and Interventions of RCTs in Pericarditis, Post-Pericardiotomy Syndrome and Post-RF Ablation for Arrythmia

	Acute Pericarditis	Acute Pericarditis	First Re-current Pericarditis	First Re-current Pericarditis	Multiply Recurrent Pericarditis	Post Pericardiotomy Syndrome	Post Pericardiotomy Syndrome	Post Pericardiotomy Syndrome	Post Pericardiotomy Syndrome	Post RF Ablation for Recurrent AFib
Colchicine Dose	0.25-0.5 mg bid (lower dose <70 kg or intolerance)	0.5 mg bid (daily ≤70 kg or intolerance)	0.25-0.5 mg bid (lower dose <70 kg or intolerance)	0.25-0.5 mg bid (lower dose <70 kg or intolerance)	0.5 mg bid (daily ≤70 kg or intolerance)	1.5 mg/d starting POD #3	0.5 mg bid (daily <70 kg) starting POD #3 with loading dose	0.5 mg bid (daily <70 kg) starting 48-72 h pre-op	0.5 mg bid	0.5 mg bid
Trial	COPE (Imazio) 2005 [18]	ICAP (Imazio) 2013 [19]	CORE (Imazio) 2005 [20]	CORP (Imazio) 2011 [21]	CORP-2 (Imazio) 2014 [22	Finkelstein 2002 [23]	COPPS (Imazio) 2010 [24]	COPPS-2 (Imazio) 2014 [25]	Sarzaeem 2014 [26]	Deftereos 2012 [27]
	N = 120	N = 240	N = 84	N = 120	N = 240	N = 163	N = 360	N = 360	N = 216	N = 230
*Trial Characteristics*										
No. Centres	2	5	1	4	4	2	6	11	1	3
Enrolment period	Jan 2002 – Aug 2004	Aug 2005 – Dec 2010	Jan 2001 – Aug 2004	Aug 2005 – Apr 2009	Nov 2005 – Jan 2012	Oct 1997 – Sept 1998	n/r	Mar 2012 – Mar 2014	Jan 2013 –Jul 2013	n/r
Treatment/ Follow Up	3 months/24 months (mean)	3 months/18 months (all)/ 22 months (mean)	6 months/20 months (mean)	6 months/ 18 months (all)/ 23 months (mean)	6 months/18 months (all)/ 20 months (mean)	1 month/3 months	1 month/19 months (mean)	1 month/3 months	7 days/Hosp discharge (mean 7 days)	3 months/15 (median) months
Funding	Public	Public	Public	Public	Public	n/r	Public	Public	n/r	n/r
*Patients*	N = 120	N = 240	N = 84	N = 120	N = 240	N = 111	N = 360	N = 360	N = 216	N = 206
Mean Age (years)	57	52	54	48	49	64	66	68	60	62
% Male	45 %	60 %	35 %	53 %	50 %	73 %	67 %	69 %	72 %	70 %
BMI									26	26
Diabetes				3 %		27 %	23 %	22 %	37 %	25 %
HTN				23 %		46 %	68 %	68 %	53 %	41 %
Smoker				49 %		48 %	13 %	29 %	30 %	35 %
Dyslipidemia						42 %				
Prev MI/UA				11 %	7 %	40 %	21 %			34 % (CAD)
Prev stroke/TIA									2 %	
PVD										
CRD	(Excl Cr >2.5 mg/dL/ 221 μM)	(Excl Cr >2.5 mg/dL/ 221 μM)	(Excl Cr >2.5 mg/dL/ 221 μM)	5 % (CrCl <60 mL/min; Excl Cr >2.5 mg/dL/ 221 μM)	(Excl Cr >2.5 mg/dL/ 221 μM)		15 % (CrCl <60 mL/min; Excl Cr >2.5 mg/dL/ 221 μM)	7 % (Excl Cr >2.5 mg/dL/ 221 μM)	Excl	Excl eGFR <30 mL/min
Mean LVEF				58 %			54 %	55 %	47 %	55 %
Previous CABG				6 %	4 %		6 %	6 %	Excl	
*Medications*										
ASA and/or clopidogrel		76 % (ASA)			76 % (ASA)					
Statin										37 %
Beta-Blocker										36 %
Calcium Channel Blocker										41 %
ACE Inhibitor										54 % (incl ARB)
Diuretic										

Abbreviations: *ACE* angiotensin converting enzyme, *ACS* acute coronary syndrome, *ARB* angiotensin receptor blocker, *AFib* atrial fibrillation, *ASA* acetylsalicylic acid (aspirin), *bid* twice daily, *BMI* body mass index, *BMS* bare metal stent, *CABG* coronary artery bypass grafting, *CAD* coronary artery disease, *CHF* congestive heart failure, *chol* cholesterol, *Cr* serum creatinine concentration, *CrCl* creatinine clearance, *CRD* chronic renal disease, *DAPT* dual anti-platelet therapy, *DM* diabetes mellitus, *dL* deciliter, *eGFR* estimated glomerular filtration rate, *excl* excluded, *h* hour, *HTN* hypertension, *kg* kilogram (body weight), *LVEF* left ventricular ejective fraction, *mg* milligram, *μM* micromolar, *MI* myocardial infarction, *mL* milliliter, *N* number of patients, *no*. number, *n/r* not reported, *PCI* percutaneous coronary intervention, *POD* post-operative day, *prev* previous, *PTCA* percutaneous coronary angioplasty, *PVD* peripheral vascular disease, *RF* radiofrequency, *TIA* transient ischemic attack, *UA* unstable angina, *yrs* years

Study quality, where specified, was relatively high (Table 3). All but three trials [14, 18, 20] were blinded using placebos and in one of these three non-placebo controlled RCTs, outcome assessors were blinded [14]. Allocation was specified to be concealed in the 7 trials that reported this information [14, 15, 19, 21, 22, 24, 25]. Thirteen trials specified that intention-to-treat analysis [7, 14, 15, 17–25, 27] was used and thirteen trials indicated that they were not stopped early for benefit [7, 14–17, 19, 21–27]. Only one trial had >10 % of randomized patients with missing outcome data [23] though in two trials the proportion of excluded randomized patients was unclear [16, 26].

**Table 3:** Quality assessment of included randomized controlled trials

Trial	Follow up duration	Blinded	Concealed allocation	Intention to treat analysis	Not stopped early for benefit	<5 % Randomized Patients with Missing Outcome Data
Nidorf 2013 [14]	3 years (median) [minimum 2 years]	Outcome assessors only	Yes	Yes	Yes	Yes (0 %, 0/532)
Raju 2012 [15]	32 days (median)	Yes	Yes	Yes	Yes	No (7.3 %, 6/82)
O’Keefe 1992 [16]	5.5 months (mean)	Yes	n/r	n/r	Yes	Unclear (unsuccessful PTCA patients excluded but randomized before PTCA)
Deftereos 2013 [7]	6 months (all)	Yes	n/r	Yes	Yes	Yes (0 %, 0/222 [clinical outcomes])
Deftereos 2014 [17]	6 months (all)	Yes	n/r	Yes	Yes	Yes (1.1 %, 3/279)
Finkelstein 2002 [23]	3 months (all)	Yes	n/r	Yes	Yes	No (32 %, 52/163)
COPE 2005 (Imazio) [18]	20 months (mean)	No	n/r	Yes	n/r	Yes (0 %)
ICAP 2013 (Imazio) [19]	18 months (all)/ 22 months (mean)	Yes	Yes	Yes	Yes	Yes (0 %)
CORE 2005 (Imazio) [20]	18 months (all)/ 20 months (mean)	No	n/r	Yes	n/r	Yes (0 %)
CORP 2011 (Imazio) [28]	18 months (all)/ 23 months (mean)	Yes	Yes	Yes	Yes	Yes (0 %)
CORP-2 2014 (Imazio) [22]	18 months (all)/ 20 months (mean)	Yes	Yes	Yes	Yes	Yes (0 %)
COPPS 2010/1 (Imazio) [24, 28]	19 months (mean)	Yes	Yes	Yes	Yes	Yes (0 %)
COPPS-2 2014 (Imazio) [25]	3 months (median)	Yes	Yes	Yes	Yes	Yes (0 %)
Sarzaeem 2014 [26]	7 days (mean) (hospital discharge)	Yes	n/r	n/r	Yes	Unclear (excluded patients unable to tolerate enteral medications within 48 h post cardiac surgery)
Deftereos 2012/ 2014 [17, 27]	3 months (?all)/ 15 months (median)	Yes	n/r	Yes	Yes	No (10 %, 24/230 [AFib recurrence]; 6.1 %, 14/230 [adverse events])

Abbreviations: *AFib* atrial fibrillation, *n/r* not reported, *PTCA* percutaneous coronary angioplasty

### Quantitative data synthesis

**Fig. 2:**
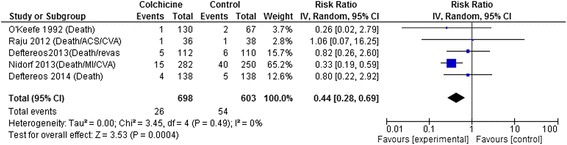
Forest Plot for Composite Cardiovascular Outcome. Individual and pooled risk ratios (RR) with 95 % confidence intervals (CI) for randomized controlled trials (RCTs) enrolling patients with cardiovascular diseases comparing colchicine to placebo or control. The pooled RRs with 95 % CI were calculated using random-effects models. Weight refers to the contribution of each study to the overall pooled estimate of treatment effect. Each square and horizontal line denotes the point estimate and 95 % CI for each trial’s RR. The diamonds signify the pooled RR; the diamond’s centre denotes the point estimate and width denotes the 95 % CI. The composite cardiovascular outcome includes the components indicated for each RCT, except for Nidorf 2013 [14] also includes cardiac arrests. Abbreviations: ACS, acute coronary syndrome; CI, confidence interval; CVA, cerebrovascular attack; IV, inverse variance; MI, myocardial infarction; revasc, revascularization

Cardiovascular Outcomes: Colchicine was associated with a >50 % reduction in the composite cardiovascular outcome (RR 0.44, 95 % CI 0.28-0.69, p = 0.0004, I^2^ = 0 %; 5 trials [1301 patients]) (Fig. 2). As indicated in Fig. 2, two of these trials reported only deaths.[16, 17]. This result was driven by the single-centre RCT in stable CAD which was the only RCT to individually demonstrate a statistically significant benefit (RR 0.33, 95 % CI 0.19-0.59, p = 0.0001; n = 532). It made up 65 % of the weighting in the pooled estimate and included mortality, myocardial infarction, stroke, and cardiac arrest in its composite outcome [14]. This trial was open label but blinded outcome assessors and treated patients for a median of 3 years [14]. Visual inspection of the funnel plot for this outcome showed no evidence of asymmetry.

**Fig. 3:**
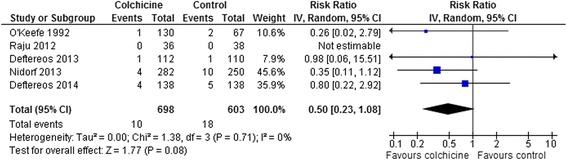
Forest Plot for All-Cause Mortality. Individual and pooled risk ratios (RR) with 95 % confidence intervals (CI) for randomized controlled trials (RCTs) enrolling patients with cardiovascular diseases comparing colchicine to placebo or control. [RCTs enrolling patients with pericarditis [18–22], post-pericardiotomy syndrome [23–26], or post-RF ablation [27] are excluded. Only two of these RCTs [24, 25] reported any deaths: 2/169 vs. 2/167 patients [24], and 6/180 vs. 2/180 [25]. Including these RCTs does not significantly change the pooled result: RR 0.74, 95 % CI 0.37-1.49, p = 0.40. The pooled RRs with 95 % CI were calculated using random-effects models. Weight refers to the contribution of each study to the overall pooled estimate of treatment effect. Each square and horizontal line denotes the point estimate and 95 % CI for each trial’s RR. The diamonds signify the pooled RR; the diamond’s centre denotes the point estimate and width denotes the 95 % CI

All-cause mortality rates were low with only 4 trials having a total of 10 deaths in the colchicine group and 18 deaths in the control group with a non-significant trend of benefit in the colchicine group (RR 0.50, 95 % CI 0.23-1.08, p = 0.08; *I*^*2*^ = 0 %) (Fig. 3). There were no differences in rates of acute coronary syndrome or non-fatal myocardial infarction (RR 0.59, 95 % CI 0.09-3.90, p = 0.58; *I*^*2*^ = 46 %; 2 trials with 14/318 vs. 34/288 events [14, 15] or post PTCA need for revascularization (RR 0.90, 95 % CI 0.62-1.30, p = 0.58; *I*^*2*^ = 0 %; 2 trials with 42/204 vs. 29/163 events [7, 16], or stroke (RR 0.41, 95 % CI 0.06-2.75, p = 0.36; *I*^*2*^ = 0 %; 2 trials with 1/318 vs. 3/288 events [14, 15]. Risk of stroke remained similar even including data from the recently published post-cardiac surgery trial that reported 2/180 vs. 1/180 patients with stroke [25]. RR 0.76, 95 % CI 0.17-3.37, p = 0.71; *I*^*2*^ = 0 %; 3 trials with 3/498 vs. 4/468 events.

**Fig. 4:**
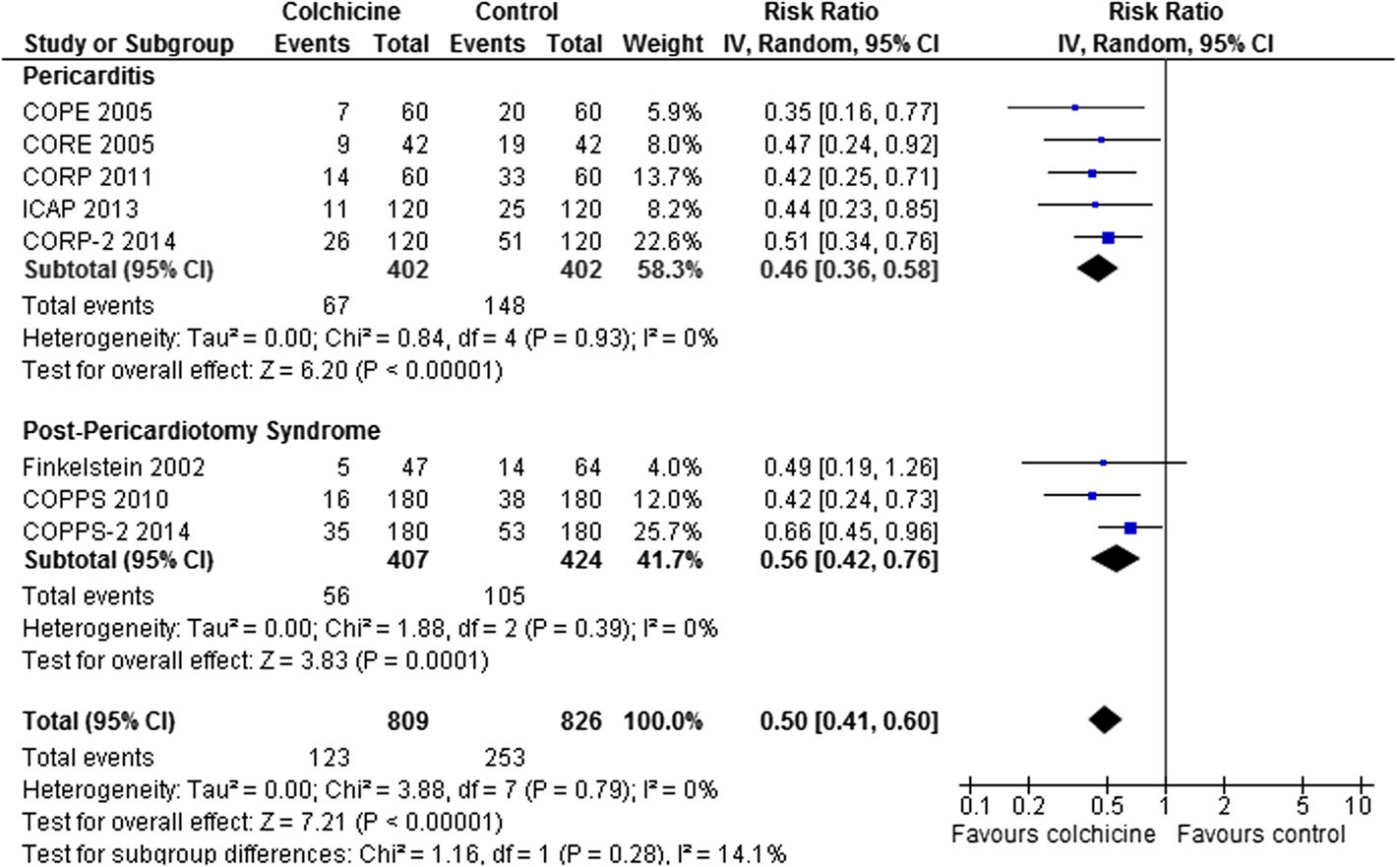
Forest Plot for Pericarditis and Post Cardiac Surgery Pericardiotomy. Individual and pooled risk ratios (RR) with 95 % confidence intervals (CI) for randomized controlled trials (RCTs) comparing colchicine to placebo or control in patients with pericarditis or post cardiac surgery pericardiotomy. The pooled RRs with 95 % CI were calculated using random-effects models. Weight refers to the contribution of each study to the overall pooled estimate of treatment effect. Each square and horizontal line denotes the point estimate and 95 % CI for each trial’s RR. The diamonds signify the pooled RR; the diamond’s centre denotes the point estimate and width denotes the 95 % CI. The decreases in risks were similar for pericarditis vs. pericardiotomy RCTs (interaction p = 0.28), and, as described in the manuscript text, also for acute vs. recurrent vs. multiple recurrent pericarditis RCTs with non-significant interaction p-values for all comparisons

Recurrent Pericarditis or Post-Pericardiotomy Syndrome: Colchicine decreased rates of recurrent pericarditis or pericardiotomy syndrome (RR 0.50, 95 % CI 0.41-0.60, p < 0.0001, I^2^ = 0 %; 8 RCTs, n = 1635) (Fig. 4). Despite different pathophysiology, this decrease was similar comparing the pooled result from the 3 post-pericardiotomy RCTs (RR 0.56, 95 % CI 0.42-0.76, p = 0.0001, I^2^ = 0 % [23–25] with the pooled result from the 5 pericarditis RCTs (RR 0.46, 95 % CI 0.36-0.58, p < 0.0001, I^2^ = 0 % [18–22]; interaction p = 0.28). The decreases were also similar comparing the pooled results from the 2 RCTs enrolling patients with acute pericarditis (RR 0.40, 95 % CI 0.24-0.66, p = 0.0004, I^2^ = 0 % [18, 19]) with the pooled results from the 3 RCTs enrolling patients with recurrent pericarditis (RR 0.48, 95 % CI 0.36-0.63, p < 0.0001, I^2^ = 0 % [20–22]; interaction p = 0.56), and similar comparing the pooled results from the 2 RCTs enrolling patients with a first recurrence of pericarditis (RR 0.44, 95 % CI 0.29-0.66, p < 0.0001, I^2^ = 0 % [20, 21] with the result of the RCT [22] enrolling patients with two or more recurrences of pericarditis (RR 0.51, 95 % CI 0.34-0.76, p = 0.0009; interaction p = 0.62) with all trials showing a roughly 50 % decrease in pericarditis recurrence. Visual inspection of the funnel plot for this outcome showed no evidence of asymmetry (results not shown).

**Fig. 5:**
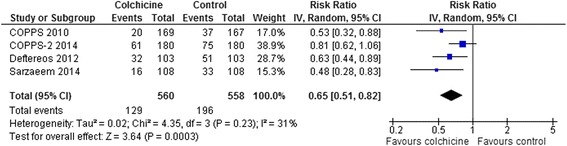
Forest Plot for Atrial Fibrillation. Individual and pooled risk ratios (RR) with 95 % confidence intervals (CI) for randomized controlled trials (RCTs) comparing colchicine to placebo or control in patients post cardiac surgery pericardiotomy, or post radiofrequency (RF) ablation for recurrent atrial fibrillation. The pooled RRs with 95 % CI were calculated using random-effects models. Weight refers to the contribution of each study to the overall pooled estimate of treatment effect. Each square and horizontal line denotes the point estimate and 95 % CI for each trial’s RR. The diamonds signify the pooled RR; the diamond’s centre denotes the point estimate and width denotes the 95 % CI

Colchicine decreased atrial fibrillation post-CABG/pericardiotomy (RR 0.63, 95 % CI 0.45-0.90, p = 0.01, I^2^ = 52 %; 3 RCTs, n = 912 [24–26] and post-ablation atrial fibrillation (RR 0.63, 95 % CI 0.44-0.89, p = 0.009; 1 RCT, n = 206 [27] by similar amounts (pooled RR 0.65, 95 % CI 0.51-0.82, p = 0.0003, I^2^ = 31 %; 4 RCTs, n = 1118) (Fig. 5).

**Fig. 6:**
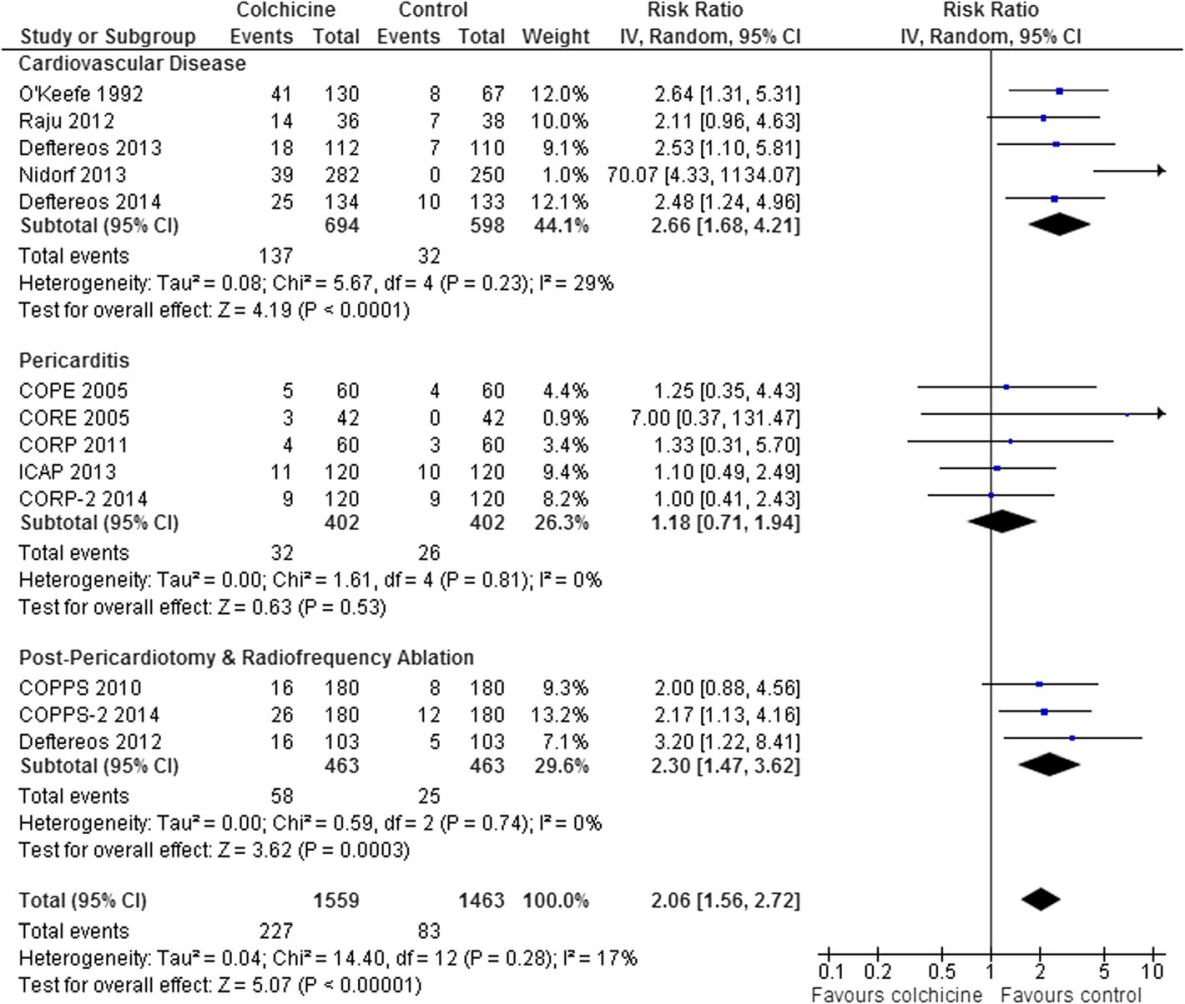
Forest Plot for Gastrointestinal Adverse Events. Individual and pooled risk ratios (RR) with 95 % confidence intervals (CI) for randomized controlled trials (RCTs) comparing colchicine to placebo or control in patients with cardiovascular diseases, pericarditis, and post pericardiotomy or radiofrequency (RF) ablation. The pooled RRs with 95 % CI were calculated using random-effects models. Weight refers to the contribution of each study to the overall pooled estimate of treatment effect. Each square and horizontal line denotes the point estimate and 95 % CI for each trial’s RR. The diamonds signify the pooled RR; the diamond’s centre denotes the point estimate and width denotes the 95 % CI. The pericarditis patient trials [18–22] which reduced the dose of colchicine for intolerance or low body weight (<70 kg) and enrolled generally younger patients (mean age 48–57), showed no significant increase in gastrointestinal side effects. This was different than the results from the other trials which showed a doubling of risk (interaction p = 0.01). Including data from all 7 RCTs that reduced the dose of colchicine for intolerance or body weight <70 kg [18–22, 24, 25], the increase in gastrointestinal adverse events was lower but still statistically significant (RR 1.56, 95 % CI 1.09-2.24, p = 0.01, I^2^ = 0 %; 7 RCTs, 1524 patients) suggesting that dose reduction by itself is not sufficient to eliminate gastrointestinal side effects. For two RCTs that reported non-diarrhea gastrointestinal side effects separately from the diarrhea side effects we assumed that the 5/130 vs. 4/67 patients with nausea or vomiting and 0/130 vs. 1/67 patients with dyspepsia were different than the 36/130 vs. 3/67 patients with diarrhea [16] and the 6/103 vs. 3/103 patients with nausea were different than the 10/103 vs. 2/103 patients with diarrhea [27]. Results are similar if one assumes that these events occurred in the same patients for these 2 RCTs (overall RR 2.11, 95 % CI 1.54-2.89, p < 0.0001, I^2^ = 26 %; 216/1559 [13.9 %] vs. 77/1463 [5.3 %])

Adverse Events: The most common adverse event was diarrhea. Pooled results from all RCTs that reported rates of diarrhea and other gastrointestinal side effects demonstrated a two-fold increase (RR 2.06, 95 % CI 1.56-2.72, p < 0.0001; 13 RCTs, 227/1559 [14.6 %] vs. 83/1463 [5.7 %]) with some heterogeneity (I^2^ = 17 %) (Fig. 6). The pericarditis patient trials [18–22] which reduced the dose of colchicine for intolerance or low body weight (<70 kg) and enrolled generally younger patients (mean age 48–57), showed no significant increase in gastrointestinal side effects. This was different than the results from the other trials which showed a doubling of gastrointestinal side effects (interaction p = 0.01). However, including data from all 7 RCTs that reduced the dose of colchicine for intolerance or body weight <70 kg [18–22, 24, 25], the increase in gastrointestinal side effects was smaller but still statistically significant (RR 1.56, 95 % CI 1.09-2.24, p = 0.01, I^2^ = 0 %; 7 RCTs, 74/762 [9.7 %] vs. 46/762 [6.0 %]), suggesting that dose reduction by itself is not sufficient to eliminate gastrointestinal side effects. Increases in other adverse events were infrequent and not statistically different between groups: neuromuscular side effects including myalgia, myopathy, neuritis, or neuropathy (RR 1.65, 95 % CI 0.86-3.15, p = 0.13, I^2^ = 0 %; 9 RCTs, 23/1192 [1.9 %] vs. 12/1191 [1.0 %] [7, 14, 15, 17, 19, 21, 22, 24, 25]; rash or puritis (RR 1.22, 95 % CI 0.24-6.12, p = 0.81, I^2^ = 0 %; 3 RCTs, 4/416 [1.0 %] vs. 2/355 [0.6 %] [14–16]; alopecia (RR 1.59, 95 % CI 0.36-6.97, p = 0.54, I^2^ = 0 %; 7 RCTs, 4/1103 [0.4 %] vs. 2/1100 [0.2 %] [7, 14, 19, 21, 22, 24, 25, 27]; elevated liver enzymes (RR 1.44, 95 % CI 0.51-4.09, p = 0.49, I^2^ = 0 %; 8 RCTs, 9/1009 [0.9 %] vs. 6/1006 [0.6 %] [7, 17, 19, 21, 22, 24, 25, 27] there were no reported cases of liver failure]; and myelotoxicity (0/507 [0.0 %] vs. 0/503 [0.0 %] [7, 17, 24, 27]. Serious or life-threatening adverse events were not reported in any trial.

**Fig. 7:**
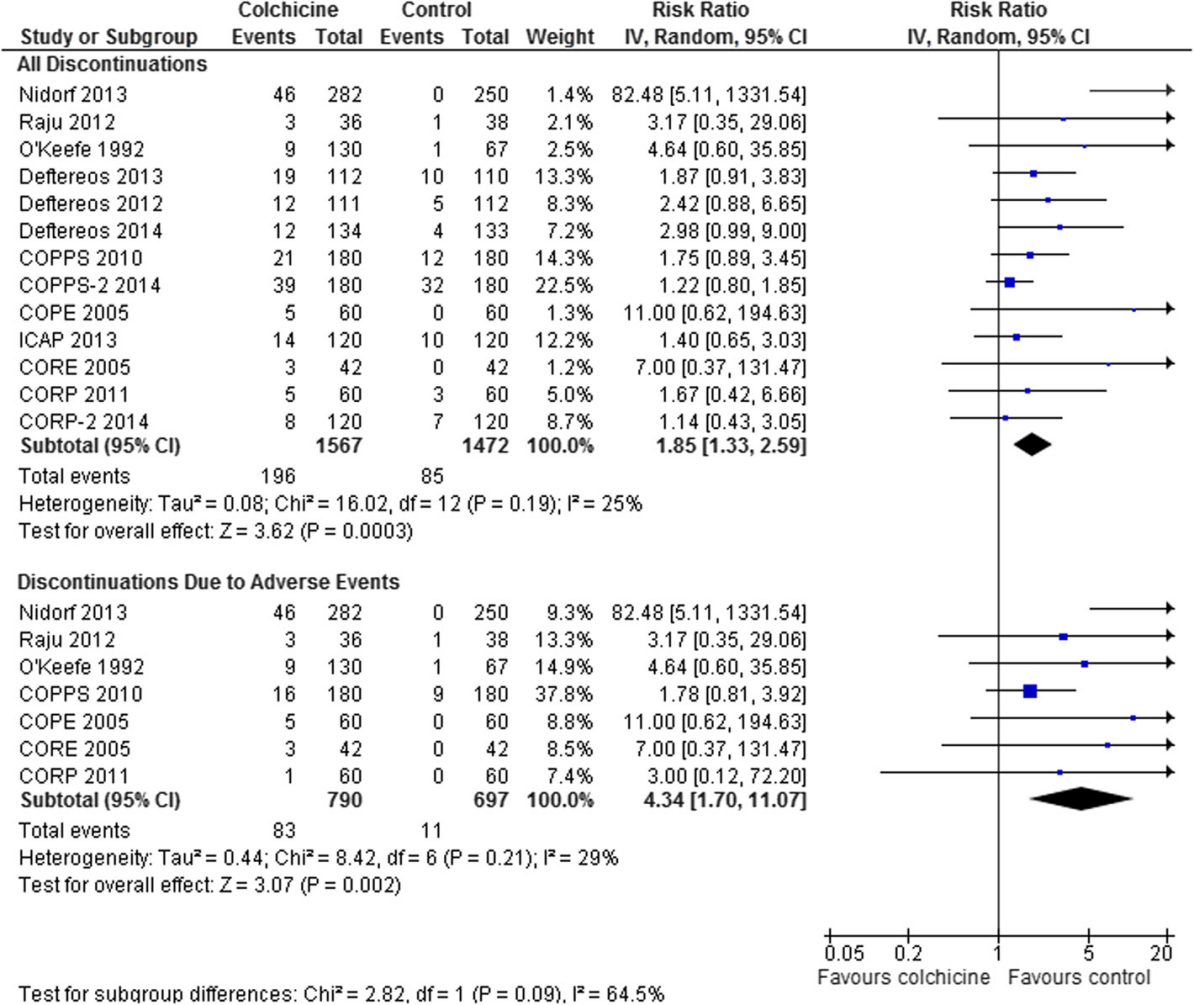
Forest Plot for All Medication Discontinuation and Discontinuation Due to Side Effects. Individual and pooled risk ratios (RR) with 95 % confidence intervals (CI) for randomized controlled trials (RCTs) comparing colchicine to placebo or control in patients with various cardiac conditions. The pooled RRs with 95 % CI were calculated using random-effects models. Weight refers to the contribution of each study to the overall pooled estimate of treatment effect. Each square and horizontal line denotes the point estimate and 95 % CI for each trial’s RR. The diamonds signify the pooled RR; the diamond’s centre denotes the point estimate and width denotes the 95 % CI. Including only data from the 7 RCTs that reduced the dose of colchicine for intolerance or body weight <70 kg [18–22, 24, 25], the rates of medication discontinuation were still increased: discontinuation overall, RR 1.40, 95 % CI 1.04-1.89, p = 0.03, I^2^ = 0 %; 7 RCTs, n = 1524; and discontinuation due to adverse events, RR 2.22, 95 % CI 1.08-4.56, p = 0.03, I^2^ = 0 %; 4 RCTs, n = 684. There were no differences in medication discontinuation overall or discontinuation due to adverse events between subgroups of cardiovascular disease, pericarditis, and post-pericardiotomy or radiofrequency ablation patient trials (interaction p = 0.16-0.18) (results not shown)

Treatment discontinuation overall (RR 1.85, 95 % CI 1.33-2.59, p = 0.0003, I^2^ = 25 %; 13 RCTs, 196/1567 [12.5 %] vs. 85/1472 [5.8 %]) and due to adverse events (RR 4.34, 95 % CI 1.70-11.07, p = 0.002, I^2^ = 29 %; 7 RCTs, 83/790 [10.5 %] vs. 11/697 [1.6 %]) was higher with colchicine (Fig. 7). This was the case even including data only from the 7 RCTs that reduced the dose of colchicine for intolerance or body weight <70 kg [18–22, 24, 25]: discontinuation overall, RR 1.40, 95 % CI 1.04-1.89, p = 0.03, I^2^ = 0 %; 7 RCTs, 95/762 [12.5 %] vs. 64/762 [8.4 %]; and discontinuation due to adverse events, RR 2.22, 95 % CI 1.08-4.56, p = 0.03, I^2^ = 0 %; 4 RCTs, 25/342 [7.3 %] vs. 9/342 [2.6 %]. There were no differences in medication discontinuation overall or discontinuation due to adverse events between subgroups of cardiovascular disease, pericarditis, and post-pericardiotomy or radiofrequency ablation patient trials (interaction p = 0.16-0.18) (results not shown).

## Discussion

As suggested by this meta-analysis, in different populations of patients with established cardiovascular disease, colchicine reduces the composite cardiovascular outcome by approximately 60 %. Although the pooled results are consistent between trials with no heterogeneity, the estimates are dominated by one open-label RCT (though with blinded outcome assessors) enrolling patients with stable coronary artery disease [14]. Our meta-analysis also demonstrates that colchicine reduces the rates of pericarditis recurrence, post-pericardiotomy syndrome, and atrial fibrillation post cardiac surgery or radiofrequency ablation by about 50 %. These pooled results are supported by numerous RCTs providing consistent results. Pooled results from all RCTs demonstrated that side effects, primarily diarrhea and other gastrointestinal symptoms, are increased contributing to higher medication discontinuation rates compared to placebo/control groups; however, no serious adverse events were reported.

Our systematic review and meta-analysis is the first to include RCTs testing the effect of colchicine in all cardiac diseases and is the largest and most comprehensive published to date. Previous reviews have included only RCTs enrolling patients with pericarditis, or the post pericardiotomy syndrome [30–34], and even the most recent of these did not include the most recently published post pericardiotomy [25] or atrial fibrillation [25–27] prevention RCTs. A protocol for a systematic review of colchicine for secondary prevention of cardiovascular disease [35] has been published by the Cochrane Collaboration.

Plausible mechanisms have been proposed to explain the apparently beneficial effects of colchicine for prevention of cardiovascular events [36]. Colchicine has been shown to inhibit neutrophil chemotaxis, ingress and activation within a pro-inflammatory environment. These anti-inflammatory effects appear to be important for preventing pericarditis recurrence; however, activated neutrophils are also present in atherosclerotic plaques and appear to play a key role in the transformation of a stable to an unstable plaque. By suppressing neutrophils, colchicine may play a role in stabilizing plaques and preventing fissuring or rupture that can result in the clinical manifestations of acute coronary ischemia or stroke. It is reasonable to speculate that colchicine has greater potential for action in stable coronary disease, where inflammatory mechanisms play a major role in disease progression, and that it may have less potential to provide any benefit in acute coronary syndromes in which atherothrombosis predominates. This may explain the large benefit observed in the only RCT that evaluated colchicine in patients with stable CAD [14], and lower or absent benefits observed in RCTs trying to prevent angioplasty induced vascular injury [16], stent-related disease [7] and acute plaque instability [15], which are different cardiovascular disease entities with unique pathophysiology. Numerous additional trials evaluating the use of colchicine in patients with stable cardiovascular disease, acute coronary syndrome, atrial fibrillation, and post operatively are listed on trial registries suggesting that more data regarding colchicine’s effectiveness in specific cardiovascular conditions will be forthcoming (Table 4). In addition several other trials of inflammation reduction in secondary prevention of cardiovascular events are currently underway, some with non-specific anti-inflammatory agents (like methotrexate), and others with specific anti-inflammatory approaches (such as cannakinumab) [37].

**Table 4:** Trial Registration Numbers of Ongoing Trials

Stable Cardiovascular Disease
ACTRN12614000093684 - The LoDoCo2 Trial: A randomised controlled trial on the effect of low dose Colchicine for secondary prevention of cardiovascular disease in patients with established, stable coronary artery disease. This study is not yet recruiting. Sponsor: Heart Research Institute of Western Australia/Aspen Pharmacare Australia. Target enrolment: 3000 patients.
NCT02153983 - Effects of Colchicine in Non-Diabetic Adults With Metabolic Syndrome. This study is currently recruiting participants. Sponsor: Eunice Kennedy Shriver National Institute of Child Health and Human Development (NICHD). Target enrolment: 100 patients.
NCT02162303 - Colchicine in Vascular Inflammation Assessed With PET Imaging (COLPET). This study is currently recruiting participants. Sponsor: Montreal Heart Institute. Target enrolment: 106 patients.
Acute Coronary Syndrome
NCT01906749 - Colchicine for Acute Coronary Syndromes (COACS). This study is currently recruiting participants. Sponsor: Maria Vittoria Hospital. Target enrolment: 500 patients.
NCT01936285 - Colchicine in ST-elevation Myocardial Infarction. This study is currently recruiting participants. Sponsor: G. Gennimatas General Hospital. Target enrolment: 75 patients.
NCT02095522 - COlchicine Improve EnDothElial Function in Non ST Elevation Myocardial Infarction Patients (CODEN). This study is not yet open for participant recruitment. Sponsor: Tel-Aviv Sourasky Medical Center. Target enrolment: 100 patients.
Percutaneous Intervention
NCT01709981 - Anti-inflammatory Effects of Colchicine in PCI. This study is currently recruiting participants. Sponsor: New York University School of Medicine. Target enrolment: 400 patients.
Post-Operative
ISRCTN72835417. COlchicine for the preVention of postopErative atrial fibRillation in patients undergoing Coronary Artery By-pass Grafting (COVER CABG). Completed. Sponsor: Catholic University of the Sacred Heart-Rome (Italy). Target enrolment: 320 patients.
ACTRN12613001345774 - Colchicine for the Primary Prevention of Atrial Fibrillation after Cardiac Surgery: A Double Blind Placebo Randomised Controlled Trial. Recruiting. Sponsor: Barwon Health - The Geelong Hospital. Target enrolment: 520 patients.
NCT01266694 - Cochicine Treatment for Post- Operative Pericardial Effusion (POPE2). This study has been completed. Sponsor: French Cardiology Society. Target enrolment: 199 patients.
NCT01985425 - Colchicine For Prevention of Perioperative Atrial Fibrillation in Patients Undergoing Thoracic Surgery Pilot Study (COP-AF Pilot) This study is currently recruiting participants. Sponsor: McMaster University. Target enrolment: 100 patients.
NCT02122484 - Colchicine in Coronary Artery Bypass Graft (CABG). This study is ongoing, but not recruiting participants. Sponsor: G.Gennimatas General Hospital. Target enrolment: 75 patients.
NCT02177266 - Colchicine to Prevent Post-Pericardiotomy Syndrome and Atrial Fibrillation. This study is not yet open for participant recruitment. Sponsor: Mayo Clinic. Target enrolment: 242 patients.
Chronic Atrial Fibrillation
NCT01755949 - Impact and Time Course of Colchicine Therapy on C-reactive Protein Elevation in Chronic Atrial Fibrillation and Post AF Ablation. This study is currently recruiting participants. Sponsor: Mayo Clinic. Target enrolment: 60 patients.

**Table 5:** Description of Excluded RCTs

Excluded Trial	Patient Inclusion	Number of Patients/Centres	Intervention	Control	Follow Up Duration	Outcomes	Reason for Exclusion
Prevention of Post-Op Afib							
Saenen et al. Eur Heart J 2013 [Abstract] [38]	Post CABG	40/single	Peri-operative Colchicine	Placebo	1 hour post CABG	Right Atrial Appendage Biopsy Pathology	Only pathology data provided
Nidorf & Thompson Am J Card 2007 [39]	Stable CAD	64/single	Colchicine	Control	4 weeks	C-reactive protein	Not randomized
Freed et al. Am J Card 1995 [40]	Post PTCA	5/single	Cochicine + Enalapril + Lovastatin	n/a	5 months	Death, MI, revasc.	Not randomized
Rab et al. JACC 1991 [41]	Post BMS PTCA	29/single	Colchicine + gluco-corticoids	Gluco-corticoids	4 months	Coronary artery aneurysm	Not randomized
Judkins et al. Heart Lung Circ 2011 [Abstract] [42]	Stable CAD	50/single	Colchicine	Control	6 months	C-reactive protein; flow mediated dilation	Cross over RCT
Luo & Yang. Hong Kong Med J 2001 [Abstract] [43]	Acute stroke	n/a	Colchicine, Cyclo-phosphamide plus Magnesium	Control	n/a	n/a	Combined intervention
Xu et al. West China Med J 1999 [Chinese] [44]	Acute stroke	64/single	Colchicine and Cyclo-phosphamide	Control	3 months	Neurological outcomes	Combined intervention
Liu et al. Chin J Geriat Cardiovasc Cerebrovasc Dis 2002 [Chinese] [45]	Acute stroke	325/multi	Colchicine and Cyclo-phosphamide	Control	3 months	Neurological outcomes, serum enolase, adverse events	Combined intervention

Abbreviations: *BMS* bare metal stent, *CABG* coronary artery bypass grafting, *CAD* coronary artery disease, *MI* myocardial infarction, *n/a* not available, *PTCA* percutaneous coronary angioplasty, *RCT* randomized controlled trial

This meta-analysis demonstrated that side effects were minimal; however, the short duration of therapy and follow up in most of the included RCTs meant that primarily early side effects, best described as intolerance that leads to early discontinuation of the medication, were recorded. In applications requiring only short term use such as pericarditis and atrial fibrillation prevention there is a need to develop preparations that address (mostly gastrointestinal) intolerance. In contrast, the use of colchicine for primary or secondary prevention of cardiovascular events will require longer term use. Based upon the experience in the treatment of familial Mediterranean fever, the incidence of late side effects of very long term continuous use of colchicine doses of up to 2–3 mg/day appear infrequent [2, 4]; however, these incidences of late side effects mostly relate to the use in younger people without vascular disease who are not on statins or at risk of multiple drug use and renal or hepatic dysfunction over years. Long-term studies in people with cardiovascular disease will be important in proving the safety of long-term lower dose therapy in this population.

### Study limitations

Although we used rigorous systematic review and meta-analytic methods consistent with PRISMA guidelines including a reproducible and comprehensive literature search strategy, clearly defined inclusion criteria, citation review, data abstraction, and quality assessment of individual studies, and a pre-defined analysis plan, we pooled results from studies enrolling patients with a variety of cardiac diseases. Except in the case of patients with pericarditis, most of the RCTs enrolled patients with related but non-identical cardiovascular diseases (e.g. single or at most two RCTs enrolled patients with stable CAD, acute coronary syndrome or acute stroke, post PTCA, etc.). This illustrates the paucity of RCT data in patients with cardiovascular diseases, and limits the ability to draw definitive conclusions regarding the effectiveness of colchicine in non-pericarditis cardiac patients. However, we minimized pooling data from disparate RCTs by including only RCTs with similar characteristics for specific outcomes. For example, for the primary outcomes, only patients in the cardiovascular disease RCTs were included in the composite cardiovascular event or mortality analyses, and only patients in the pericarditis RCTs had recurrent pericarditis events. Similarly the low number of non-gastrointestinal adverse events limits the ability of our systematic review to rule out increases in other, potentially more serious, adverse events since the upper limit of the 95 % confidence intervals of the RR’s of the non-gastrointestinal adverse event pooled analyses all included increased risks that would be clinically significant.

## Conclusions

In a wide range of patients with established cardiovascular disease colchicine reduces the composite rate of adverse cardiovascular outcomes. Furthermore, colchicine is associated with a significant reduction in the rates of recurrent pericarditis, post-pericardiotomy syndrome, and peri-procedural atrial fibrillation following cardiac surgery and atrial fibrillation ablation. This suggests that the diverse anti-inflammatory effects of colchicine appear to have benefits in a wide range of cardiovascular diseases. These data support the need for future RCTs of colchicine especially for the secondary prevention of cardiovascular disease.

## Abbreviations

ACE: angiotensin converting enzyme
ACS: acute coronary syndrome
ARB: angiotensin receptor blocker
AFib: atrial fibrillation
ASA: acetylsalicylic acid (aspirin)
bid: twice daily
BMI: body mass index
BMS: bare metal stent
CABG: coronary artery bypass grafting
CAD: coronary artery disease
CHF: congestive heart failure
chol: cholesterol
CI: confidence interval
Cr: serum creatinine concentration
CrCl: creatinine clearance
CRD: chronic renal disease
CVA: cerebrovascular attack
DAPT: dual anti-platelet therapy
DM: diabetes mellitus
dL: deciliter
eGFR: estimated glomerular filtration rate
excl: excluded
h: hour
HTN: hypertension
IV: inverse variance
kg: kilogram (body weight)
LVEF: left ventricular ejective fraction
mg: milligram
μM: micromolar
MI: myocardial infarction
mL: milliliter
N: number of patients
no.: number
n/r: not reported
PCI: percutaneous coronary intervention
POD: post-operative day
prev: previous
PTCA: percutaneous coronary angioplasty
PVD: peripheral vascular disease
RCT: randomized controlled trial
revasc: revascularization
RF: radiofrequency
TIA: transient ischemic attack
UA: unstable angina
yrs: years
